# Rare-earth doped strontium hexaferrite nanocomposites for enhanced electromagnetic shielding and heavy metal remediation

**DOI:** 10.1038/s41598-025-31949-4

**Published:** 2025-12-27

**Authors:** Rania Ramadan, Mai M. El-Masry

**Affiliations:** 1 Materials Science Lab. (1), Physics Department, Faculty of Science, Cairo University, Giza, Egypt; 2 Basic Science Department, Higher Engineering Institute, Thebes Academy, Cairo, Egypt

**Keywords:** Sm and Dy-doped hexaferrites, Optical conductivity, Dielectric constant, Lead ions removal, Kinetics isotherm, Chemistry, Engineering, Environmental sciences, Materials science

## Abstract

The textile and dyeing industries contribute significantly to environmental pollution through wastewater containing hazardous heavy metals, resulting in around 1.7 million deaths annually. Effective treatment methods are urgently needed. This study explores samarium (Sm) and dysprosium (Dy) doped M-type strontium hexaferrites (M-SrHFs), which improve lead adsorption due to their high surface reactivity and ion-exchange capacity. Additionally, these materials exhibit tunable electromagnetic properties, making them suitable for low-frequency attenuation or signal-conditioning applications. Dy-doped samples show optimal EMI performance at 20 MHz (RL ≈ − 0.15 dB), while co-doped variants exhibit strong absorption at 3–4 MHz with large skin depths, ideal for low-frequency industrial applications. The dual functionality of these doped M-SrHFs—effective heavy metal removal and low-frequency attenuation—highlights their potential as sustainable, multifunctional materials for environmental and electronic industry applications. This research bridges materials science and environmental engineering, offering an integrated solution for pollution control and electromagnetic compatibility.

## Introduction

The large volume of wastewater from the textile and dyeing industries poses a significant threat to the environment^1^. The textile and dyeing industries produce significant volumes of wastewater, posing serious environmental threats. Water pollution contributes to about 1.7 million deaths annually, according to the WHO. Therefore, developing safe, eco-friendly methods to remove impurities from water is crucial. Key treatment techniques include filtration, adsorption, titration, coagulation, and precipitation^2,3^. Traditional methods used for disinfecting wastewater include membrane filtering, adsorption, and chlorination. Heavy metals pose serious dangers Even at extremely low concentrations, potentially contributing to various human health issues, including cancers, allergies, lung conditions, asthma, and damage to the brain and central nervous system^3^. The textile and dyeing industries produce large amounts of wastewater that threaten the environment. Researchers are exploring innovative techniques for effective wastewater treatment to help preserve the environment. Scientists are particularly interested in nanomaterials due to their versatile properties^4^. The effectiveness of the adsorption process is highly dependent on the size and shape of the nanoparticles. The environment faces significant threats from the large volume of wastewater produced by the textile and dyeing industries. Recently, researchers have expressed significant interest in developing innovative techniques that show great potential for environmental preservation, particularly in wastewater treatment. Photo-catalysts have been extensively employed to treat various hazardous wastes, including chemical effluents containing dyes^5^. Nanoscience and nanotechnology are becoming increasingly recognized as effective solutions to many modern challenges. In fact, these nanoparticles tend to alter their structure in response to magnetic fields^6^. The high Curie temperature (720 K), elevated saturation magnetization, significant hysteresis loss, and large magneto-crystalline anisotropy make M-type strontium hexaferrites (M-SrHFs) increasingly sought after in the scientific and technological community. Strontium hexaferrites (SrFe_12_O_19_) have gained attention for their favorable optical, electrical, and magnetic properties in recent years. Additionally, SrFe_12_O_19_ is notable for its heat and corrosion resistance, making it suitable for a variety of applications^7^. The application primarily relies on the composition, magnitude, and orientation of the magnetic moment. Furthermore, the electrical and magnetic properties of M-SrHFs significantly improve when a small number of rare-earth elements (such as Sm^3^⁺, Gd^3^⁺, Er^3^⁺, Ce^3^⁺, Eu^3^⁺, Nd^3^⁺, Dy^3^⁺, etc.) is substituted into SrFe_12_O_19_ nanoferrites^8^. Microstructural factors can help explain the dielectric properties observed in ferrites. These dielectric characteristics depend on several factors, such as the synthesis method, sintering duration, temperature, grain structure, and the chemical composition of additives. Analyzing these dielectric characteristics provides important information for potential applications.

In the current study, Fe^3^⁺ magnetic ions are distributed across five hexagon-shaped sub-lattices, classified under the space group P63/mmc. Within these sub-lattices, the ions located at the 4(f1) and 4(f2) sites exhibit spins in one direction (↓), while those at the 12(k), 2(a), and 2(b) sites exhibit spins in the opposite direction (↑)^9,10^. Methods such as co-precipitation, solution combustion, hydrothermal procedures, and ceramic processes are employed to create mesoporous strontium hexaferrite (M-SrHFs). Among these, the auto combustion method stands out as a promising strategy for generating the desired phase^11^. In this study, we investigated the effects of structural, morphological, magnetic, as well as electric of M-type nano ferrites, specifically Sr_(1-x)_RELi _x_Fe_12_O_19_ (where x = 0.05 and RE represents Sm and/or Dy). We also explored the auto-combustion method for synthesizing RE-ion substituted SrFe_12_O_19_, using urea as a fuel. The novelty of this study focuses on examining the effects of structural, morphological, magnetic, and electrical properties of M-type nanoferrites, specifically Sr(1-x)RE_x_Fe_12_O_19_, where x = 0.05 and RE represents rare earth elements such as Samarium (Sm) and/or Dysprosium (Dy)^12^. The introduction of RE ions into the strontium hexaferrite structure is anticipated to significantly modify its properties. The specific concentration of x = 0.05 was selected to investigate the initial effects of RE substitution without causing drastic changes to the hexaferrite structure.

Additionally, the study investigates the auto-combustion method for synthesizing RE-ion substituted SrFe_12_O_19_ using urea as a fuel. This choice is noteworthy because urea is an abundant, cost-effective, and environmentally friendly fuel that enhances the auto-combustion process. Utilizing urea can lead to a more controlled and efficient synthesis of the desired nanoferrites.

## Experimental technique

### Sample preparation

**Fig. 1:**
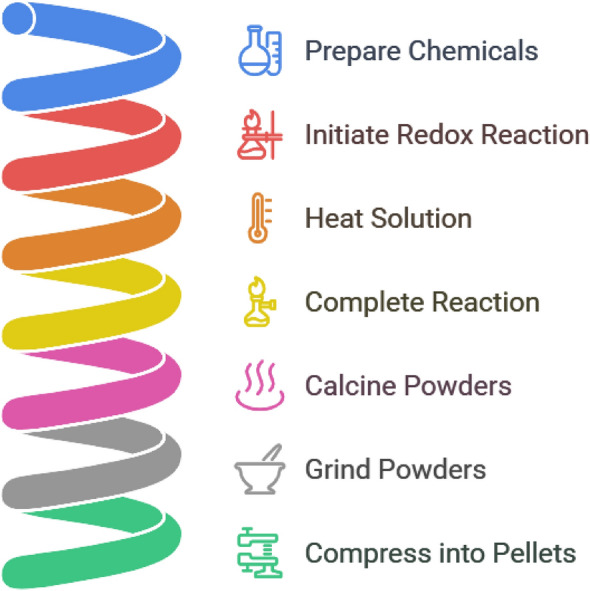
Synthesis of nanosamples.

The process of synthesizing nano samples with specific chemical formulas using the flash auto-combustion technique as shown in Fig. 1. The samples produced include Sr_(1-x)_ RExFe_12_O_19_ (where x = 0.05 and RE represents Sm and/or Dy). The methodology involves the careful combination of raw ingredients, thermal treatment, and grinding to achieve the desired phase of the materials. The raw materials used for the synthesis include Strontium Nitrate (Sr(NO_3_)_2_), Samarium Nitrate Hexahydrate (Sm(NO_3_)_3_·6H_2_O), Dysprosium Nitrate Hexahydrate (Dy(NO_3_)_3_·6H_2_O), Iron Nitrate, and Urea. These ingredients were combined in accordance with their stoichiometric ratios to ensure the correct chemical composition of the final products. A slight quantity of deionized water was added to the mixture of raw ingredients. The solution was then stirred at a temperature of 80 °C. This step was crucial for eliminating any fumes and ensuring the formation of a homogeneous powder. After stirring, the resultant solution was allowed to cool, resulting in the formation of a fine powder. This powder is the precursor for the subsequent sintering process. The produced powder was subjected to grinding to achieve a uniform particle size. Following this, the powder was sintered at a temperature of 1000 °C for a duration of 6 h. This thermal treatment is essential for achieving the necessary crystalline phase of the materials.

### Characterization techniques for nanosamples

Various characterization techniques are employed to analyze the crystalline structure, morphology, magnetic properties, and dielectric characteristics of Nano samples. The methodologies discussed include X-ray diffraction (XRD), energy dispersive X-ray spectroscopy (XPS), Fourier transform infrared spectroscopy (FTIR), Ultraviolet and visible (UV–Vis) spectroscopy, vibrating sample magnetometry (VSM), and LCR testing. Each technique provides valuable insights into the physical and chemical properties of the samples, contributing to a comprehensive understanding of their behavior and potential applications.

#### Crystalline structure analysis

The crystalline structure of the samples was examined using an X’Pert Pro diffractometer. This technique allows for the identification of the crystalline phases present in the samples and provides information on their structural properties.

#### Morphology examination

To analyze the morphology of the samples, energy dispersive X-ray spectroscopy (EDX) was conducted in conjunction with a scanning electron microscope (SEM). This combination enables detailed imaging and elemental analysis, allowing for a thorough understanding of the sample’s surface characteristics.

#### Fourier transform infrared spectroscopy (FTIR)

FTIR measurements were conducted at ambient temperature using a PerkinElmer spectrometer (Model: Spectrum 2, USA). The measurements covered a wavenumber range of 4000 cm⁻^1^ to 300 cm⁻^1^, providing insights into the functional groups and molecular interactions within the samples.

#### UV–Vis spectroscopy

The UV–Vis spectra associated with the produced Nano samples have been obtained by the JASCO Corp. V-570 spectrometer, Rev. 1.00. This technique is essential for understanding the optical properties related to the nanosamples, including their absorption and transmission characteristics.

#### Magnetic properties analysis

The magnetic hysteresis loops were measured at room temperature using a vibrating sample magnetometer (VSM) on powder samples. The saturation magnetization (Ms) and remanent magnetization (Mr) are reported in emu/g (specific magnetization), which is mass-normalized and independent of the powder’s packing density. Coercivity (Hc) is reported in Oersted (Oe). Given the random orientation of particles in the powder sample, demagnetization effects are not applied, and the measured properties are considered intrinsic to the material.

#### Preliminary low-frequency electromagnetic characterization

The LCR Hi-tester (Model: HIOKI, JAPAN) has been used to investigate the dielectric characteristics of the cylindrical sample’s pellets (10 mm diameter × 1–2 mm thickness) were prepared by uniaxial pressing of the synthesized powders with at 6 tons/cm^2^, followed by sintering at 1200 °C for 4 h. Silver electrodes were applied to both faces for electrical contact. around 300 K, within a frequency range of mHz to MHz.

### Impact of lead adsorption using concentration-specific lead solutions

**Fig. 2:**
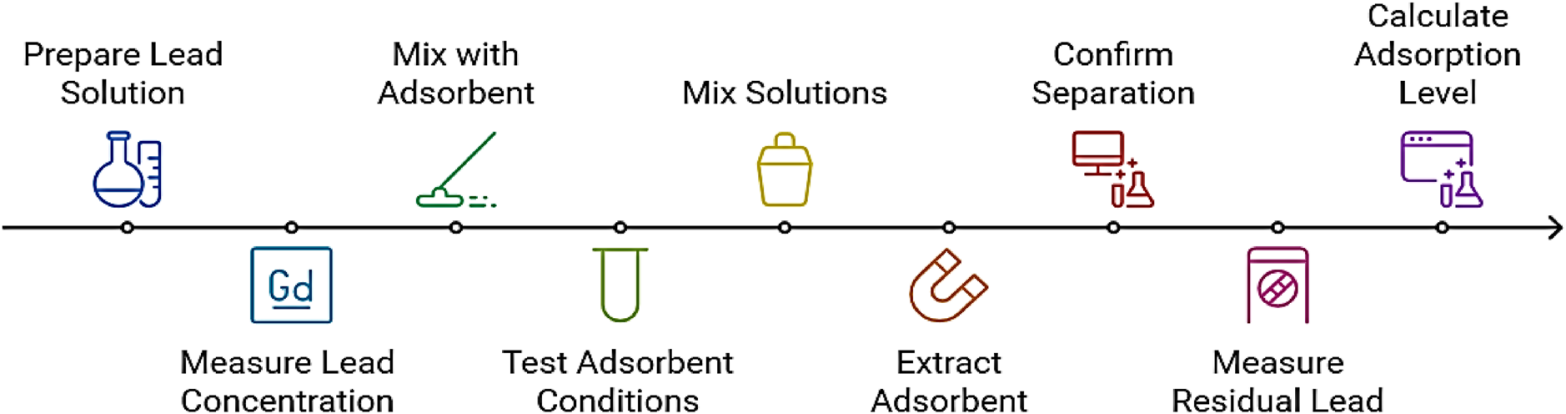
Lead adsorption process.

This experiment was designed to investigate the adsorption of lead from a concentration-specific lead solution using prepared adsorbent samples Fig. 2. The study employs atomic absorption spectrometry (AAS) to measure lead concentrations and evaluates the effectiveness of the adsorbent under varying conditions such as concentration, time, temperature, and pH. The results demonstrate the efficiency of the adsorbent in removing lead from the solution, highlighting the importance of optimizing various parameters for enhanced adsorption. A concentration-specific lead solution was prepared to examine the impact of lead adsorption using the prepared samples. The concentration of lead within the solution was measured using atomic absorption spectrometry (AAS). This lead-based solution was then mixed with the adsorbent. Multiple samples of the lead solution were prepared at different concentrations. The adsorbent was tested under various conditions, including different concentrations, times, temperatures, and pH values. A shaker was employed to mix the liquid combinations at a consistent speed. After a short mixing period, a magnet was positioned next to the solution to extract the adsorbent. We observed that nearly all particles of the adsorbent were successfully separated from the water due to their strong magnetic properties. To evaluate the effectiveness of the produced materials in removing lead, a batch experiment was conducted under various conditions, including different pH levels. A weight of 0.1 g of the prepared samples was added to a standard lead nitrate solution with a concentration of 2 ppm. The pH of the solution was adjusted between 2 and 8 to assess its impact on lead removal. After stirring the mixture for one hour, the solutions were filtered using a 0.2 µm syringe filter. The final concentration of lead (Pb II) was determined using atomic absorption spectroscopy (Zeenit 700P, Analytical Jena).

This was confirmed by weighing the adsorbent before as well as after the adsorption process and washing it. Atomic absorption spectrometry was employed to measure the residual lead content in each separate solution. The level of adsorption for each sample has been calculated using the given equation (Ramadan, 2024a) (1):

1 $$\text{Removal efficiency }\left(\mathrm{R}\right)\mathrm{\%} =\frac{\left({C}_{o}-C\right)}{{C}_{o}}\times 100\%$$

where: c_o_= initial concentration of lead (ppm), c_e_= equilibrium concentration of lead (ppm).

Additionally, the amount of adsorbent material q_e_ was determined using the Eq. (2):

2 $${q}_{e}=\frac{\left({C}_{o}-C\right)V}{m}$$

where (M) represents the mass of the adsorbent in grams per liter. Various parameters, including time, temperature, and pH, were investigated to enhance adsorption.

### Results and discussions

#### XRD

The structural characterization of the synthesized samarium (Sm) and/or dysprosium (Dy) doped strontium hexaferrites, with nominal compositions Sr₀.₉₅Sm₀.₀₅Fe₁₂O₁₉, Sr₀.₉₅Dy₀.₀₅Fe₁₂O₁₉, and Sr₀.₉Sm₀.₀₅Dy₀.₀₅Fe₁₂O₁₉, was conducted using X-ray diffraction (XRD) at room temperature.

**Fig. 3:**
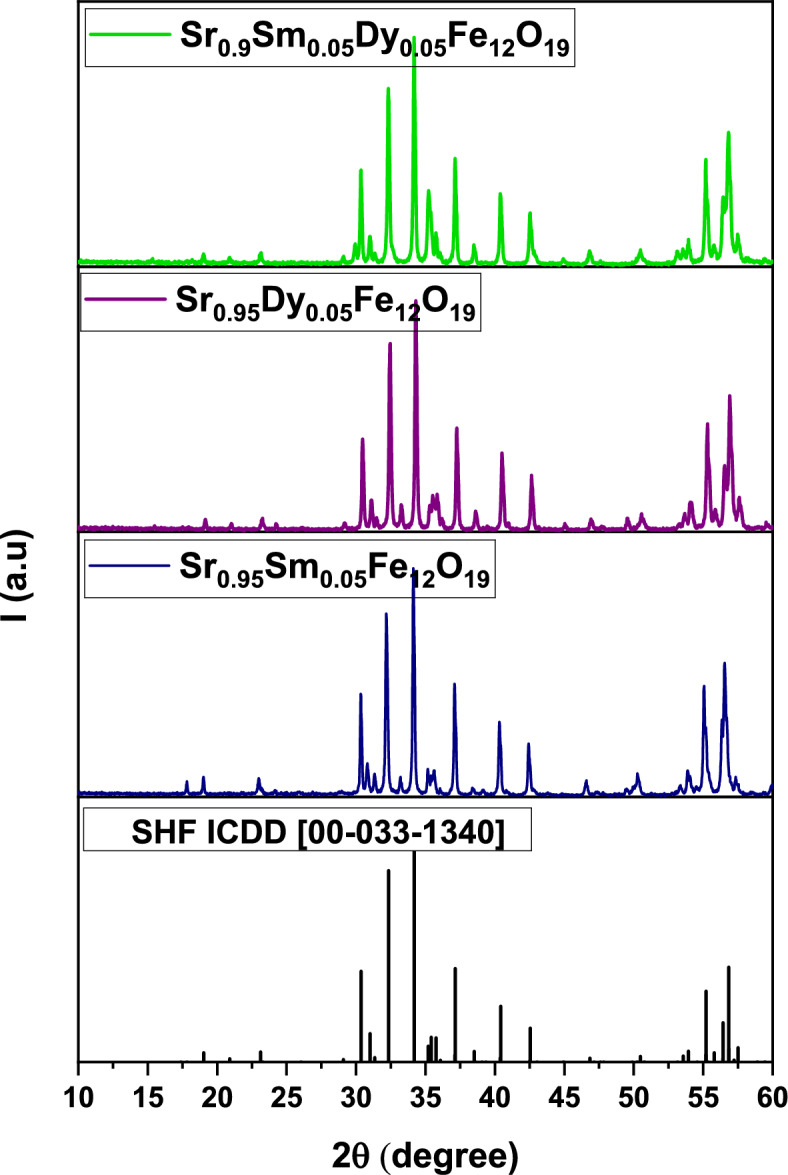

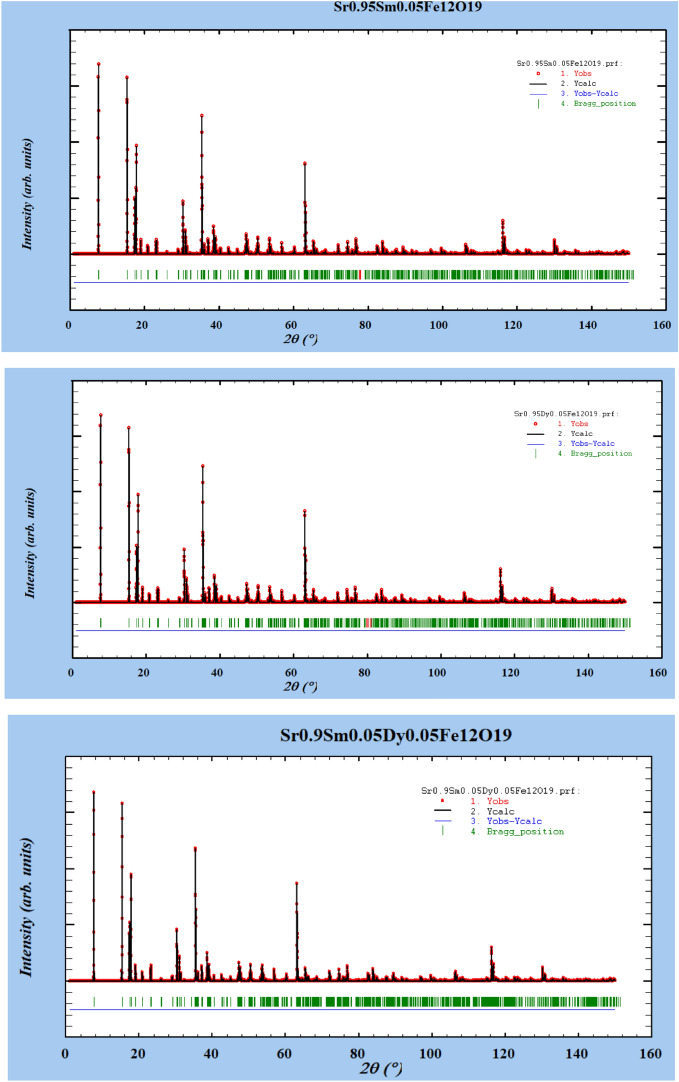
(**a**) XRD of prepared samples. (**b**) Rietveld refinement of XRD data for the prepared samples.

Figure 3a presents the XRD patterns for all prepared samples. All the observed diffraction peaks are indexed to the standard pattern for M-type strontium hexaferrite (JCPDS card No. 84-1531), confirming the successful formation of a single-phase magneto plumbite structure with the P6₃/mmc space group. No secondary or impurity phases were detected within the instrument’s resolution limit, indicating that the Sm^3^⁺ and Dy^3^⁺ ions have been effectively incorporated into the hexaferrite lattice, substituting for the Sr^2^⁺ sites. The influence of Sm^3^⁺ and/or Dy^3^⁺ substitution on the microstructural parameters was investigated. The average crystallite size (D) was estimated from the most intense peak using Scherrer’s formula^13^ (3):

3 $$D=\frac{0.9\lambda }{\beta cos\theta }$$

where λ is the X-ray wavelength (1.5406 Å for Cu-Kα), θ is the Bragg diffraction angle, and β is the full width at half maximum (FWHM) of the diffraction peak in radians. The calculated crystallite sizes are listed in Table 1. The values range from 35 to 38 nm, with the co-doped sample (Sr₀.₉Sm₀.₀₅Dy₀.₀₅Fe₁₂O₁₉) exhibiting the largest crystallite size. This suggests that the co-doping of Sm and Dy influences the crystal growth dynamics, leading to a slight increase in crystallite size compared to the singly doped samples.

**Table 1:** Microstructural parameters derived from XRD analysis for the prepared Sr hexaferrite samples.

Sample	Crystallite size (nm)	Microstrain (ε × 10^–4^)	Dislocation density (δ × 10^–4^)(nm^−2^)	Theoretical density (ρ_x_) (g/Cm^3^)	Bulk density (ρ_b_) (g/Cm^3^)	Porosity %
Sr_0.95_Dy_0.05_Fe_12_O_19_	37	12	7	5.11	3.6	29.5
Sr_0.95_Sm_0.05_Fe_12_O_19_	35	10	7.30	5.10027	3.62106	29
Sr_0.9_Sm_0.05_ Dy_0.05_ Fe_12_O_19_	38	15	6.92	5.13	3.6	30

The X-ray density (ρₓ) was calculated using the formula:

4 $${\rho }_{x}=\frac{z\times M}{{N}_{A}\times V}$$

where Z is the number of formula units per unit cell (Z = 2 for M-type hexaferrite), M is the molecular weight of the composition, Nₐ is Avogadro’s number, and V is the volume of the unit cell. As summarized in Table 1, the value of ρₓ increases with the incorporation of Dy^3^⁺ and/or Sm^3^⁺ dopants. Since the unit cell volume (V) remains relatively constant, this increase is directly attributed to the higher atomic weights of Dy (162.50 g/mol) and Sm (150.36 g/mol) compared to the Sr^2^⁺ (87.62 g/mol) they replace, thereby increasing the molecular weight (M) of the compound^14^.

In contrast, the bulk density (ρ_b_) was measured experimentally using the mass and dimensions of the pelletized samples according to the relation (3):

where *m*, *r*, and *h* are the mass, radius, and thickness of the pellet, respectively. The bulk density values, provided in Table 1, are consistently lower than the corresponding X-ray densities. This is a common feature in ceramic materials and indicates the presence of intrinsic porosity.

The percentage porosity (P%) was subsequently calculated using the relation^3,3^.

5 $$P\%=\left(1-\frac{{\rho }_{b}}{{\rho }_{x}}\right)\times 100\%$$

The porosity values, also presented in Table 1, fluctuate around 30% among all samples. These non-linear variations in porosity are well-documented for M-type hexaferrites and are influenced by a combination of factors, including the synthesis technique, sintering conditions, and the doping-induced changes in crystallite size and densification behavior (5).

Furthermore, the dislocation density (δ), which quantifies the number of defects in the crystal lattice, was determined using the formula^16^ (6):

6 $$\delta =\frac{1}{{D}^{2}}$$

where D is the average crystallite size. The calculated values are included in Table 1. A lower dislocation density generally correlates with improved mechanical strength. In this study, the sample with the largest crystallite size (Sr₀.₉Sm₀.₀₅Dy₀.₀₅Fe₁₂O₁₉) exhibits the lowest dislocation density, suggesting it may possess superior mechanical properties among the series.

#### Rietveld refinement of XRD data

To quantify structural changes and to verify phase purity, Rietveld refinement of the powder XRD patterns was performed using FullProf. The refinements used the M-type SrFe_12_O_19_structure (space group P 63/m m c) as the starting model. The background (6-term polynomial), scale factor, hexagonal lattice parameters (a and c), and profile parameters (U, V, W of the pseudo-Voigt profile) were refined sequentially. Occupancies on the Sr (2d) site were fixed to the nominal synthesis values (Sr_0.95_Sm_0._ Fe_12_O_19_); occupancy refinement was attempted only under site-sum constraints to avoid strong correlations with the scale factor. Oxygen occupancies were not refined because laboratory XRD is not sufficiently sensitive to reliably determine small oxygen deficiencies. The final refined lattice parameters, unit-cell volumes, and phase fractions are summarized in Table 2. The observed and calculated patterns together with the different curves are shown in Fig. 3b. These Rietveld results confirm that the samples are single-phase M-type Sr-hexaferrites within the detection limits of our XRD measurements; small systematic changes in a and c upon Sm/Dy doping are consistent with the substituted ionic radii and the structural perturbations reported in the literature.

**Table 2:** Rietveld results of unit-cell volumes, and phase fractions for the prepared Sr hexaferrite samples.

Sample	a (Å)	c (Å)	V (Å^3^)	Phase fraction (%)
Sr₀.₉₅Sm₀.₀₅Fe₁₂O₁₉	a = 5.8860	c = 23.0400	691.2794	100
Sr₀.₉₅Dy₀.₀₅Fe₁₂O₁₉	a = 5.8842	c = 23.0420	690.9	100
Sr₀.₉Sm₀.₀₅^Dy^₀.₀₅Fe₁₂O₁₉	a = 5.8820	c = 23.0350	690.1903	100

### FTIR

**Fig. 4:**
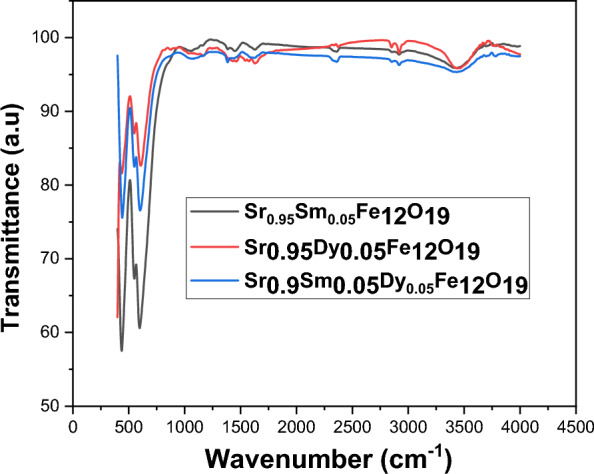
FTIR measurements of prepared nanoparticles in the range 400–4000 cm^−1^.

The FT-IR spectra for the compositions of Sr_0.95_Sm_0.05_Fe_12_O_19_, Sr_0.95_Dy_0.05_Fe_12_O_19_, and Sr_0.9_Sm_0.05_Dy_0.05_Fe_12_O_19_ were recorded in the wavenumber range of 4000 to 300 cm⁻^1^ at room temperature. As shown in Fig. 4, each sample’s spectrum displays two distinct and strong absorption bands, which indicate the strength of the magneto-plumbite structure. The observed absorption bands correspond to the vibrational modes associated with the octahedral and tetrahedral sites within the ferrite structure. Specifically, the strong vibrational bands are linked to two wavenumber ranges: 400–450 cm⁻^1^ for the octahedral sites, and 500–600 cm⁻^1^ for the tetrahedral sites^17^. These bands represent the characteristic oscillations of the respective sites, thereby confirming the structural integrity and phase purity of the synthesized nanohexaferrites. The presence of these strong absorption bands in the FTIR spectra serves as a crucial indicator of the material’s magnetic properties and overall performance in various applications. FTIR spectroscopy is an invaluable tool for verifying single-phase ferrites, providing insight into their molecular structure and potential contaminants^18^. The analysis of investigated nanohexaferrites demonstrates the effectiveness of FTIR in identifying key vibrational modes that are essential for understanding the material’s properties. Future studies may build on these findings to explore the implications of varying compositions on the performance of ferrite materials in practical applications. The effects of Samarium (Sm) and Dysprosium (Dy) substitution in hexaferrite samples, emphasizing the variations in peak positions and their implications for the crystal structure. The findings suggest that Sm^3^⁺ and Dy^3^⁺ ions are incorporated at the Sr^2^⁺ sites of the hexagonal lattice. The charge imbalance (Sm^3^⁺/Dy^3^⁺ replacing Sr^2^⁺) is compensated primarily by partial reduction of Fe^3^⁺ to Fe^2^⁺ and/or the creation of oxygen vacancies, which slightly modifies the Fe^2^⁺/Fe^3^⁺ ratio and influences the magnetic interactions all without altering the overall crystal structure of the material. The synthesis of hexaferrite is confirmed by the presence of characteristic peaks in the vibrational spectrum, specifically around 440 cm⁻^1^ and 550 cm⁻^1^, which are attributed to the stretching vibrations of metal-oxygen bonds. The positions of these peaks show slight variations in the Sm and/or Dy-substituted samples. Therefore, RE^3^⁺ replaces the Sr^2^⁺ ions, without any changes to the crystal structure composition^19^. The calcination process and its impact on the absorption peaks of carbon dioxide groups and nitrate ions. The analysis highlights the role of urea as reductants and nitrate ions as oxidants during the redox reaction, as well as the influence of moisture on the spectral characteristics of the samples. After calcination, there were no peaks detected for carbon dioxide groups or nitrate (NO_3_) ions, indicating that the redox reaction has concluded. During this reaction, urea acted as reductants while nitrate ions served as oxidants^20^. This finding suggests that the chemical changes occurring during calcination effectively remove these specific ions from the sample. Additionally, the samples have absorbed moisture, which is reflected in the spectrum by a frequency band starting at 1630 cm^−1^. This band corresponds to the bending mode of water molecules^21^. The impact of substituting Sm^3+^ and/or Dy^3+^ ions on the band positions of tetrahedral and octahedral clusters, specifically focusing on how variations in Fe^3+^–O^2−^ bond lengths affect absorption peak positions. The findings reveal significant shifts in these absorption peaks, highlighting the occupancy of different sites by the respective ions. The incorporation of RE^3+^ ions into the crystal structure is expected to preferentially occupy octahedral sites, leading to a noticeable shift in the absorption peaks associated with these locations. As RE^3+^ ions occupy the octahedral positions, the corresponding absorption peaks are observed to move to higher frequency regions. This shift indicates changes in the local environment surrounding the Fe^3+^–O^2−^ bonds, which are influenced by the presence of RE^3+^ ions.

### Morphology study

**Fig. 5:**
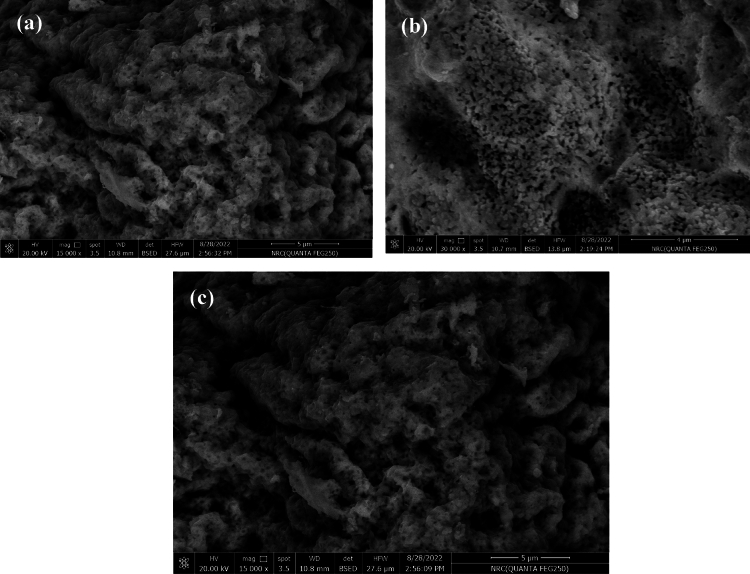
(**a**–**c**) SEM micrographs and histograms for (**a**) Sr_0.95_Sm_0.05_Fe_12_O_19_, (**b**) Sr_0.95_Dy_0.05_Fe_12_O_19_, (**c**) Sr_0.9_Sm_0.05_Dy_0.05_Fe_12_O_19_.

The scanning electron microscope (SEM) images of the produced sample compositions are displayed in Fig. 5a–c. It is evident that the fragments aggregate and exhibit porosity. Due to their primary particle associations and magnetic properties, the hexaferrite particles are highlighted in the micrographs. The fine powders tend to clump together into larger masses. Additionally, the samples demonstrate a tendency to form relatively regular hexagonal platelets in a parallel and multilayer arrangement. Reports indicate that the auto-combustion method, which involves a fuel reaction using citric acid, leads to crystallization rather than obtaining crystals from a solution of nanoparticles related to hexaferrites. This is attributed to the brief duration and high temperature required for the reaction to complete.

### Optical properties

**Fig. 6:**
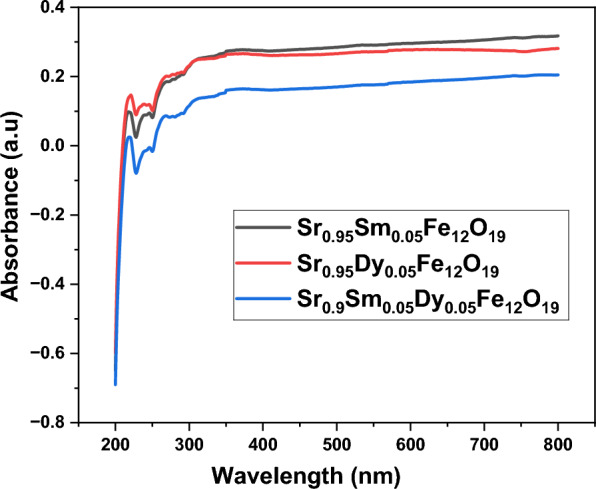
UV–Vis absorbance spectrum of prepared nanoparticles.

Absorbance spectra with wavelengths ranging from 200 to 600 nm were used to evaluate the optical properties of the prepared powders; the resulting spectra are shown in Fig. 6. An atom can change between its ground state and a highly excited state when the energy of an incident photon surpasses the atom’s band gap energy. An electron crosses the optical band gap between the valence and conduction bands when it is excited by photons. As the band gap energy decreases, electrons migrate from the valence band to the conduction band (Eg). Understanding the electronic characteristics of materials and their possible uses in a variety of industries, including photovoltaics, photodetectors, and optoelectronics, depends on this phenomenon. The efficiency and functionality of semiconductor materials are largely dependent on the electrons’ freedom to move between these bands when exposed to incident photons. In summary, the absorbance spectrum analysis highlights the significance of band gap energy in electron excitation and migration and offers important insights into the optical characteristics of the produced powders. The implications of these findings in real-world applications could be investigated in more detail^22^. Tauc’s relation (T-region) is used in this study to estimate the optical band gap energy Eq. (7)^23,24^.

7 $$\left( {\alpha {\mathrm{h}}\nu } \right)^{{\mathrm{n}}} = {\mathrm{A}}\left( {{\mathrm{h}}\nu - {\mathrm{E}}_{{\mathrm{g}}} } \right)$$

where A is the constant, α is considered as the absorption coefficient, and n indicates the type of optical transition (n = 2 for direct transitions and n = ½ for indirect transitions).

The findings are compared with previously reported estimates for strontium hexaferrite, providing insights into the factors influencing band gap shifts.

**Fig. 7:**
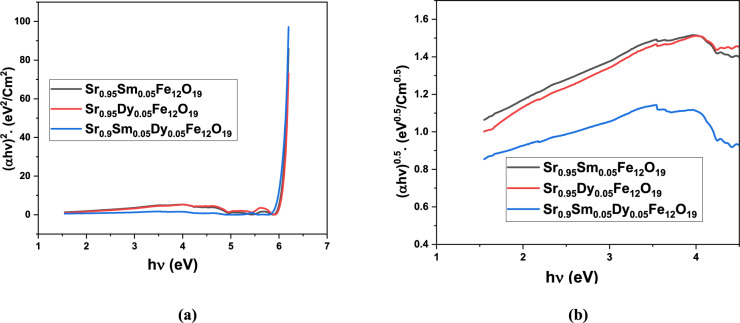
(**a**) (αhυ)^2^ and (**b**) (αhυ)^0.5^ versus hυ of prepared nanoparticles.

The relationship between (αhv)2 and (αhv)⁰0.5 versus incident photon energy (hν) is depicted in Fig. 7a,b. M-type strontium hexaferrite (SrFe₁₂O₁₉) is known to be an indirect band gap semiconductor, which aligns with our findings the indirect transition yields lower, more physically meaningful values (~ 2.15–2.32 eV) compared to the direct gap (~ 6.0 eV). The high direct gap is typical for wide-bandgap oxides and reflects transitions involving higher-energy conduction bands not involved in primary optical absorption. The observed reduction in indirect band gap with co-doping (2.15 eV) suggests:

1. Lattice strain due to ionic radius mismatch (Sm^3^⁺ = 1.08 Å, Dy^3^⁺ = 1.03 Å vs. Sr^2^⁺ = 1.44 Å) distorts Fe–O bonds, narrowing the valence/conduction band separation.
2. Possible introduction of mid-gap states from oxygen vacancies or Fe^2^⁺/Fe^3^⁺ mixed valence.
3. Enhanced visible-light absorption (λ < 570 nm), making co-doped material more suitable for photocatalytic or optoelectronic applications under solar irradiation.

These values are consistent with reported indirect band gaps for undoped SrFe₁₂O₁₉ (≈2.1–2.4 eV)^25,26^ and doped variants (down to 2.0 eV with La or Gd)^27,28^. The further reduction to 2.15 eV in co-doped sample confirms the synergistic effect of dual rare-earth substitution. A band gap of ~ 2.15–2.32 eV allows absorption of visible light (~ 560–540 nm), enabling potential use in water splitting or pollutant degradation under sunlight. Suitable for UV–visible photodetectors or sensors and not suitable for IR applications.

The obtained results using Tauc analysis confirms that Sr₀.₉Sm₀.₀₅Dy₀.₀₅Fe₁₂O₁₉ exhibits the narrowest indirect band gap (2.15 eV) among the studied samples, indicating enhanced visible-light response due to combined Sm/Dy doping. This makes it the most promising candidate for visible-light-driven applications such as photocatalysis or photodetection.

The refraction index represents a key property of materials that significantly impacts optics and materials science. It is directly linked with the local electric field and the electronic polarizability of ions within the material Eq. (8)^29,30^:

8 $$\mathrm{n}= \frac{\left(1+\mathrm{R}\right)}{\left(1-\mathrm{R}\right)}+ \sqrt{\frac{4\mathrm{R}}{{\left(\mathrm{R}-1\right)}^{2}}-{\mathrm{K}}^{2}},$$

where R represents reflectance and K is symbol for the extinction coefficient.

**Fig. 8:**
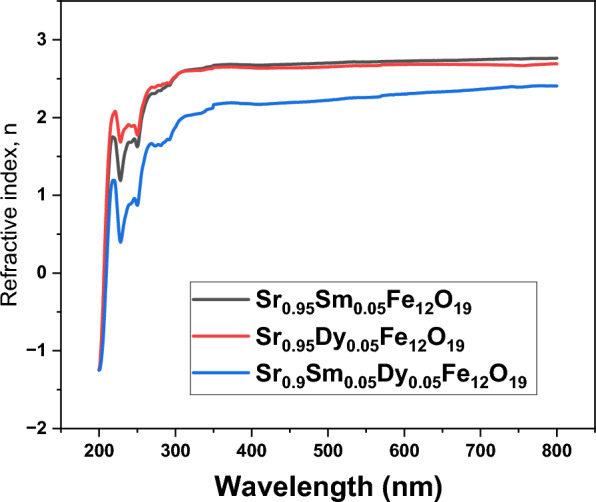
Refractive index variation regarding prepare nanoparticles with wavelength.

The analysis of the refractive index (n) of SrFe_12_O_19_ as influenced by the addition of Samarium (Sm) and/or Dysprosium (Dy). The graphical representation of the refractive index plotted against wavelength (λ) is illustrated in Fig. 8. The findings indicate a significant increase in the refractive index value due to the incorporation of these elements, which is linked to enhanced intermolecular interactions. This increase can be attributed to stronger intermolecular chemical and physical interactions between the ions, which enhance the density of SrFe_12_O_19_ and subsequently raise its refractive index. So, the study highlights the importance of chemical composition in determining the optical properties of materials, particularly in the context of magnetic ferrites like doping Sr-hexaferrite. Further research could explore the implications of these findings for applications in optics and materials science.

When studying the electronic states in materials, a crucial parameter to consider is the optical conductivity, denoted as ${\upsigma }_{\mathrm{opt}.}$. The following formula was used to determine the optical conductivity Eq. (9):

9 $${\sigma }_{\mathrm{opt}.}= \frac{\alpha \mathrm{nc}}{4\pi },$$

α refers to the absorption coefficient, and n represents the refractive index of the samples.

**Fig. 9:**
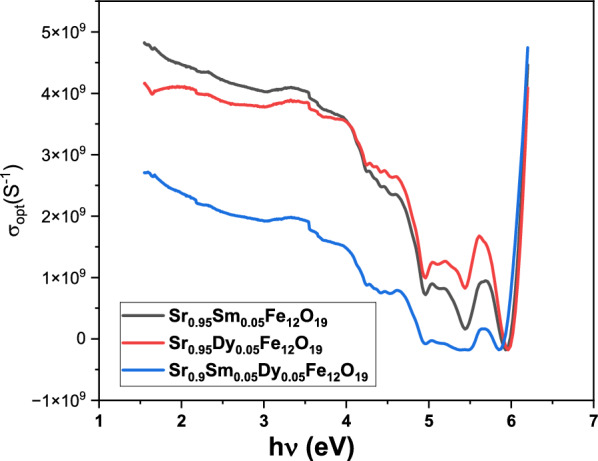
Optical conductivity variation of prepared nanoparticles with photon energy.

In the samples under investigation, Fig. 9 illustrates how optical conductivity (σ_opt_) changes with photon energy (hν). It has been observed that adding Sm and/or Dy to SrFe_12_O_19_ enhances its optical conductivity. This increase is attributed to the formation of additional band gap levels, which facilitates the migration of charge carriers from the valence band to the conduction band.

An understanding of the behaviour of electrical charge carriers in materials can be enhanced by examining the complex dielectric constant. The imaginary part associated with the dielectric constant gives information about the amount of energy absorbed by the dielectric material from the electric field due to dipole movement. In contrast, the real part related to the dielectric constant indicates the extent to which the speed of light is reduced within the material^31^. The expressions mentioned earlier were originally used to determine both the real as well as imaginary components of the dielectric constant.

The dielectric constant consists of two parts:

The real part, represented by εr, is given by the Eq. (10):

10 $$\varepsilon_{{\mathrm{r}}} = {\text{ n}}^{2} \, {-}{\text{ k}}^{2} ,$$

The imaginary part, represented by εi, is given by the Eq. (11):

11 $$\varepsilon_{{\mathrm{i}}} = {\text{ 2nk}},$$

**Fig. 10:**
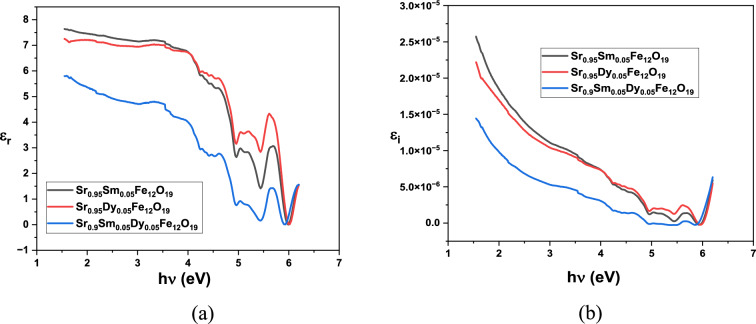
(**a**) Real dielectric constant, (**b**) imaginary dielectric constant of the prepared nanoparticles.

In these equations, ε_r_ corresponds to the real component associated with the dielectric constant, while ε_i_ corresponds to the imaginary component. Figure 10a,b illustrates the real part (ε_r_) related to the dielectric constant and the imaginary part (dielectric loss) (ε_i_) for the studied samples as a function of hv. The dielectric response of the samples has been enhanced by the addition of Sm ions. Notably, the Sr_0.95_Dy_0.05_Fe_12_O_19_ sample exhibited higher dielectric values, with the dielectric constant increase from 6 to 8. This enhancement in dielectric properties reflects the impact of the doped Sm ions. These increased dielectric values resulted from interfacial polarizations across the conductor–insulator boundary.

### Magnetic study

Doping involves substituting or adding ions into the crystal lattice of the host material. In the case of Sr hexaferrites, Dy and Sm ions can occupy specific sites within the lattice, influencing the overall magnetic interactions. The presence of these rare earth ions can enhance magnetic anisotropy and alter the exchange interactions between the iron ions in the lattice.

Dysprosium ions have a high magnetic moment and strong spin–orbit coupling, which can lead to increased magnetic anisotropy. The introduction of Dy into the Sr hexaferrite structure can enhance the coercivity and saturation magnetization due to the alignment of Dy moments with the iron moments. This alignment can result in improved performance in applications requiring high magnetic stability.

Samarium ions also exhibit significant magnetic properties, but their effect on the magnetic behavior of Sr hexaferrites can differ from that of Dy. Sm doping can lead to a reduction in saturation magnetization due to the weaker magnetic moment compared to Dy. However, Sm can enhance the thermal stability of the magnetic properties, making it suitable for high-temperature applications.

**Fig. 11:**
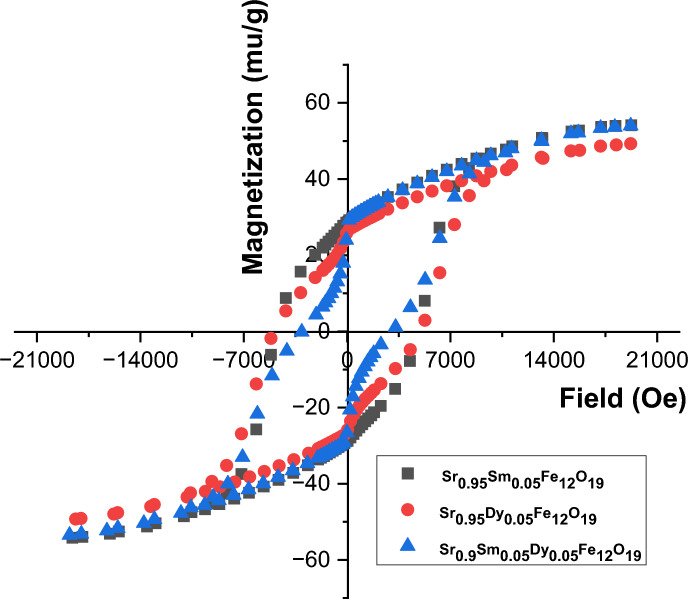
VSM of the prepared samples.

The magnetic hysteresis loop depicted in Fig. 11 reveals significant alterations in the magnetic characteristics of SHF due to the doping of Sm and/or Dy ions at the Sr lattice positions. As mentioned in Table 3. The saturation magnetization (Ms) values range from 49.293 to 54.142 emu/g, while the remanent magnetization (Mr) values fall between 28.784 and 25.971 emu/g. The coercivity (Hc) of the samples is measured to be between 4745 and 3040.7 Oe. In the hexaferrites structure, Fe ions occupy distinct lattice sites which is five with varying spin orientations, including three octahedral sites (12 k (↑), 2a (↑), 4f2 (↓)), one tetrahedral site (4f2 (↑)), in addition to one trigonal bipyramidal site (2b (↑)). The substantial contribution of these lattice sites to the magnetism of Sr- hexaferrite is attributed to super-exchange interaction coupling between Fe3 + ions via O^2−^ ions. Notably, if smaller magnetic ions replace the Fe (5 μB) site, the Ms value will gradually decrease. Conversely, if the replacement elements favor the 4f2 (↓) down-spin direction, the Ms of the material will increase. Research on M-type hexaferrites has shown that the conversion of Fe^3+^ into Fe^2+^ due to the presence of rare earth cations leads to a decrease in Ms and Mr values, as Fe^2+^ possesses a lower magnetic moment (4.89 μB) compared to Fe^3+^ (5.91 μB). Additionally, the potential for spin canting at Fe^3+^ sub-lattice locations contributes to the reduction in Ms when Sm^3+^ is doped into SHF. Reports indicate that the substitution of Dy^3+^ cations for Fe^3+^ cations at spin-up sub-lattice sites (2a, 12k, and 2b) disrupts the balance of Fe^3+^ distribution, further lowering the Ms value. The magnetic moment associated with the Sm^3+^ ion (1.38 μB) is significantly less than that of the Fe^3+^ ion (5 μB), which also contributes to the overall decrease in magnetization of the hexaferrite. While the magnetic moment for Dy^3+^ (10.5 μB). The variations in μB suggest additional factors influencing the changes in Ms. Overall, the Ms value does not show a significant improvement with Sm replacement, likely due to the lower magnetic moment of Sm ions.

**Table 3:** Magnetic parameters of the prepared samples.

Sample	Ms(emu/g)	Mr(emu/g)	Hci(Oe)	Ms/Mr	Area(erg/g)
Sr_0.95_Sm_0.05_Fe_12_O_19_	49.291	25.971	4745	1.89	576.33 × 10^3^
Sr_0.95_Dy_0.05_Fe_12_O_19_	54.142	28.784	4899.4	1.88	611.44 × 10^3^
Sr_0.9_Sm_0.05_Dy_0.05_Fe_12_O_19_	53.733	26.039	3040.7	2.063	474.77 × 10^3^

### Reflection loss (RL)

Reflection loss refers to the attenuation or reduction in the intensity of an electromagnetic wave when it encounters a boundary between two materials with different electromagnetic properties, such as air and solid material. It is caused by the portion of the wave that is reflected at the interface rather than being transmitted into the second material. The reflection loss (RL) is calculated using the impedance mismatch between the investigated materials and free space Eq. (8)^32,33^:

12 $$\mathrm{RL}= 20 {\mathrm{log}}_{10}\left|\frac{\mathrm{Z}- {\mathrm{Z}}_{\mathrm{o}}}{\mathrm{Z}+ {\mathrm{Z}}_{\mathrm{o}}}\right|[\mathrm{dB}]$$

where, Z = Z′ − jZ′′ (complex impedance of your material).

Z_o_​ = 377Ω (characteristic impedance of free space).

**Fig. 12:**
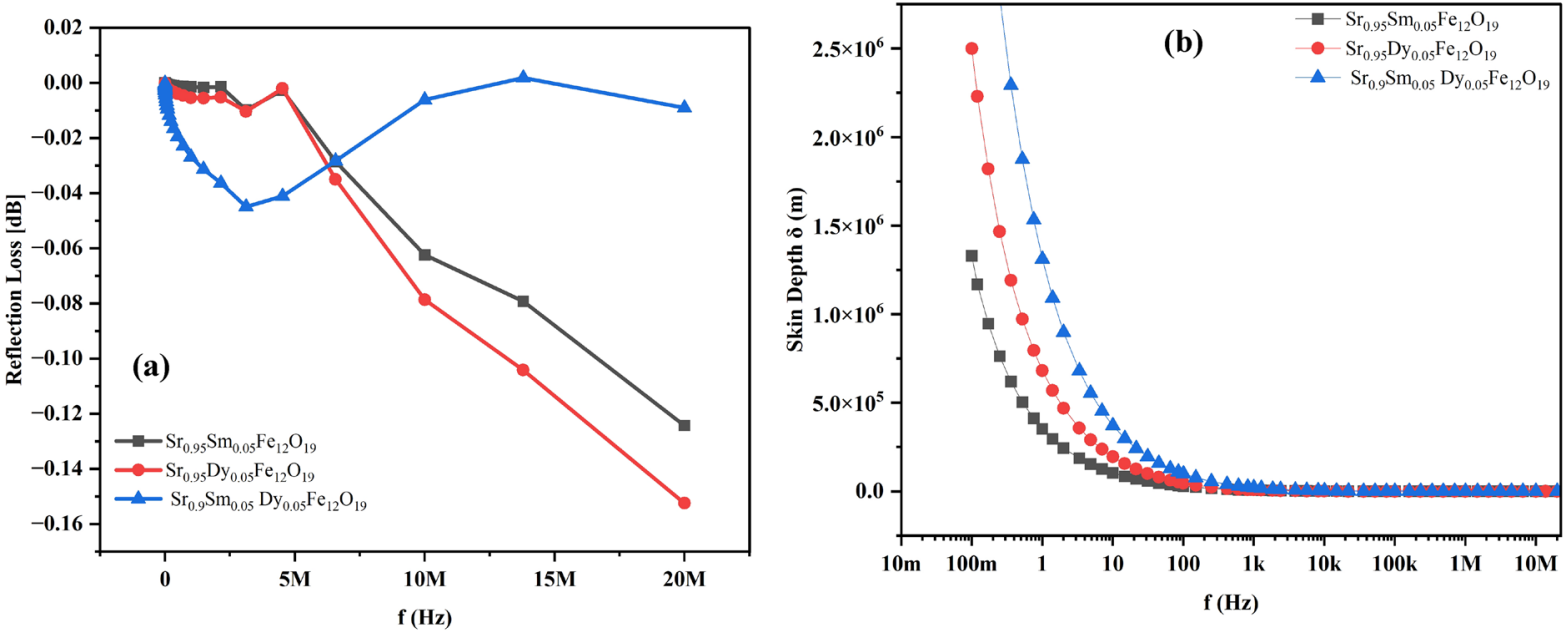
(**a**) Reflection loss (dB) (**b**) Skin depth of Sr_0.95_Sm_0.05_Fe_12_O_19_, Sr_0.95_Dy_0.05_Fe_12_O_19_, Sr_0.9_Sm_0.05_Dy_0.05_Fe_12_O_19_ nanocomposite at frequency range from 1 Hz to 20MHz.

Figure 12a illustrates the reflection loss (RL) of Sr_0.95_Sm_0.05_Fe_12_O_19_, Sr_0.95_Dy_0.05_Fe_12_O_19_, Sr_0.9_Sm_0.05_Dy_0.05_Fe_12_O_19_ nanocomposite at frequency range from 1 Hz to 20MHz. The Sm-doped sample (Sr₀.₉₅Sm₀.₀₅Fe₁₂O₁₉) exhibits low RL values (close to 0 dB) below 5 MHz, with minimal fluctuations. Above 5 MHz, RL decreases progressively, reaching approximately − 0.125 dB at 20 MHz, indicating enhanced absorption at higher frequencies. Similarly, the Dy-doped sample (Sr₀.₉₅Dy₀.₀₅Fe₁₂O₁₉) shows comparable behavior in the low-frequency range but demonstrates a more pronounced decrease in RL above 5 MHz, achieving the lowest value among the samples (− 0.15 dB at 20 MHz). This suggests that Dy doping is slightly more effective than Sm doping in promoting reflection loss at higher frequencies, potentially due to mechanisms such as ferromagnetic resonance or increased dielectric losses induced by Dy incorporation into the lattice structure.

In contrast, the co-doped sample (Sr₀.₉Sm₀.₀₅Dy₀.₀₅Fe₁₂O₁₉) displays a unique RL profile, with a distinct absorption peak centered around 3–4 MHz, reaching a modest minimum RL of − 0.045 dB. This indicates superior performance in the lower frequency range (0–5 MHz) compared to the single-doped materials. However, beyond 5 MHz, its RL increases (approaching 0 dB by 15 MHz), reflecting diminished absorption capabilities at higher frequencies. The co-doping of Sm and Dy likely induces complex interactions that modify magnetic anisotropy, permeability, and permittivity, shifting or enhancing resonance phenomena (e.g., domain wall or natural resonance) within the low-frequency regime.

Overall, the RL values across the measured spectrum (1 Hz to 20 MHz) are relatively small (maximum loss of − 0.15 dB). While these values fall short of typical microwave absorption requirements (e.g., below − 10 dB or − 20 dB in the GHz range), the observed trends are significant for tuning material properties within this specific frequency band. Single doping (Sm or Dy) enhances high-frequency absorption, with Dy showing slightly better performance, while co-doping optimizes absorption in the low MHz range (around 3–4 MHz) but compromises higher-frequency performance. These findings demonstrate the tunability of electromagnetic properties in hexaferrites through controlled rare-earth substitution, offering potential applications tailored to specific frequency ranges. Further studies, including higher-frequency analysis and correlations with structural/magnetic properties, could optimize these materials for targeted electromagnetic applications.

### Skin depth

The frequency dependence of the skin depth (δ) for three strontium hexaferrite nanocomposites Sr_0.95_Sm_0.05_Fe_12_O_19_, Sr_0.95_Dy_0.05_Fe_12_O_19_, Sr_0.9_Sm_0.05_Dy_0.05_Fe_12_O_19_ was studied over a frequency range of approximately 10 mHz to 10 MHz as shown in Fig. 12b. Skin depth is a critical parameter that quantifies the penetration depth of electromagnetic waves into a material before their amplitude decays to 1/e of the initial value. It is inversely related to the material’s ability to attenuate electromagnetic waves, making it highly relevant for applications like electromagnetic interference (EMI) shielding. The skin depth depends on the frequency (f), electrical conductivity (σ), and magnetic permeability (μ) of the material, following the relation Eq. (13)^34–36^:

13 $$\updelta = \sqrt{\frac{2}{\omega \mu \sigma }}$$

$\upomega$ is the angular frequency, $\upmu$ is the magnetic permeability, σ is the total electrical conductivity.

The results show that all three samples exhibit a pronounced decrease in skin depth as frequency increases, consistent with the theoretical relationship δ ∝ 1/f.

​. At very low frequencies (mHz range), skin depth values are exceptionally large (~ 10^6^ m), indicating deep wave penetration. As frequency increases into the Hz, kHz, and MHz ranges, skin depth decreases rapidly, reaching near-zero values in the kHz to MHz region. This demonstrates that the materials become increasingly effective at attenuating electromagnetic waves at higher frequencies.

A comparative analysis reveals differences in skin depth among the samples, particularly at lower frequencies (< 100 Hz). The Sm-doped sample (Sr_0.95_​Sm_0.05​_Fe_12_​O_19_​) consistently exhibits the smallest skin depth, indicating superior attenuation capabilities. For example, at 100 mHz, its skin depth is approximately 1.3 × 10^6^ m, compared to 1.8 × 10^6^ m for the Dy-doped sample (Sr_0.95_​Dy_0.05​_Fe_12_​O_19_) and 2.3 × 10^6^m for the co-doped sample (Sr_0.9_​Sm_0.05​_Dy_0.05_​Fe_12_​O_19_​). These trends suggest that Sm doping most effectively enhances either conductivity (σ), permeability (μ), or both, resulting in reduced skin depth. Dy doping shows intermediate performance, while co-doping yields the largest skin depth, likely due to a lower μσ product.

At higher frequencies (kHz to MHz), the skin depth values converge to very small levels for all samples, indicating their potential suitability for preliminary low-frequency electromagnetic applications in this range. However, the differences observed at lower frequencies highlight the tunability achieved through specific doping strategies. The enhanced attenuation properties of Sm-doped samples at lower frequencies underscore the significant impact of rare-earth dopant selection on tailoring the electromagnetic characteristics of these materials.

### Adsorption study

#### Effect of pH on adsorption

The prepared samples were subjected to a series of tests to evaluate its effectiveness in adsorbing Pb heavy metal ions under varying pH conditions and contact times. This was conducted through a batch experiment utilizing an aqueous solution containing dispersed Pb(II) ions. The unique properties of the nano-powder, such as its strong magnetism and small size, facilitated the physical binding of Pb ions.

**Fig. 13:**
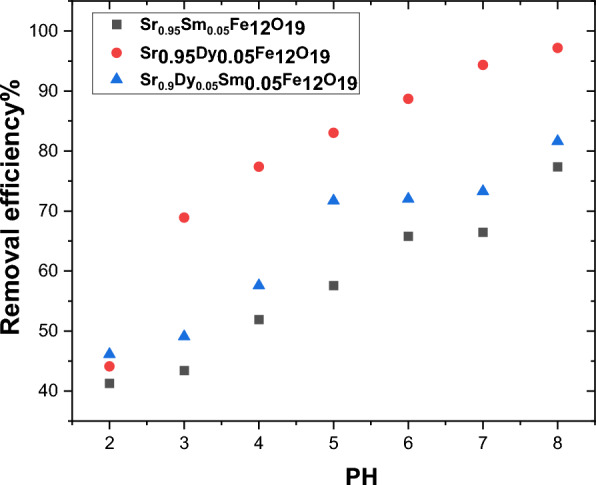
The impact of pH on the prepared samples’ Pb^2+^ removal efficiency.

The adsorption process is influenced by the chemical composition of the solution, particularly the pH value and contact time. By adjusting the pH from 2 to 8 at a constant temperature of 300 K, the study aimed to elucidate the impact of pH on the elimination of Pb^2+^ waste. A total of 20 mg of nano powder was added to the solution, and the results are depicted in Fig. 13.

The pH level plays a crucial role in determining the stability and speciation of metals during the adsorption process, as well as the molecular structure configuration. It was observed that basic media exhibited a greater capacity for Pb^2+^ adsorption compared to acidic media. At elevated pH levels, metal ions tend to form insoluble hydroxide complexes, which enhance the binding of molecules to the surfaces of the samples. This observation aligns with the conclusions drawn from Ref.^24^ the study.

The minimum adsorption of Pb ions was recorded at pH 2. This phenomenon can be attributed to the higher mobility and concentration of hydrogen ions at lower pH values, which favors the adsorption of H^+^ ions over Pb II ions. Consequently, as the pH increases, the availability of protons decreases, leading to a more neutral or negatively charged active site. The electrostatic attraction force further enhances the sorption of positively charged metal ions.

The study revealed a rapid increase in the removal efficiency of heavy metal ions as pH levels rose, with a notable change from 69 to 97% removal efficiency when the pH was adjusted from 3 to 7. Among the samples tested, Sr_0.95_Dy_0.05_Fe_12_O_19_ demonstrated the highest removal efficiency, as illustrated in Fig. 13. In contrast, Sr_0.95_Sm_0.05_Fe_12_O_19_ nanoparticles exhibited lower efficiency at pH 7, ranging from 40 to 50%. This improvement in removal efficiency can be attributed to the doping of rare earth elements, which affects the roughness, surface area, and particle size, ultimately enhancing the uptake of heavy metals.

#### Adsorption kinetics of Pb(II) ions onto nanoparticles

**Fig. 14:**
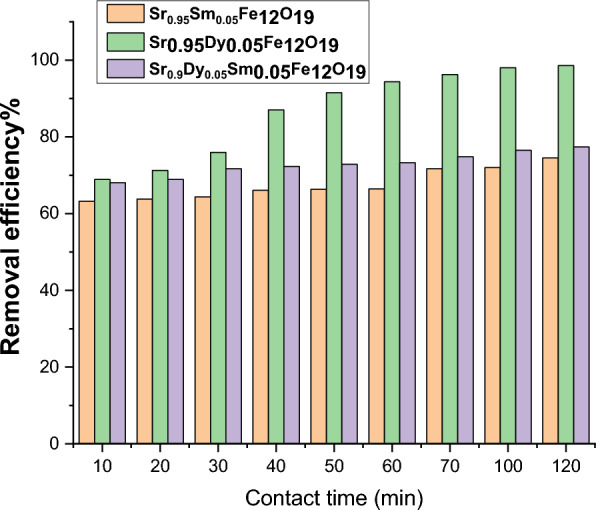
Impact of contact time on the Pb^2+^ removal efficiency prepared samples.

The adsorption kinetics of Pb(II) ions have been thoroughly investigated to determine the rate at which these ions are adsorbed onto the prepared samples. As illustrated in Fig. 14, the impact of contact duration on Pb(II) adsorption is significant. The process can be divided into two distinct phases:

1. *Initial Rapid Adsorption*: In the first half hour of contact, the removal efficiency of Pb(II) ions increases sharply. This rapid uptake can be attributed to the availability of numerous active sites on the nanoparticles, which facilitate the quick adsorption of Pb(II) ions.
2. *Equilibrium Phase*: Following the initial phase, the rate of adsorption decreases as the system approaches an equilibrium state. This decline in the rate of adsorption suggests that the active sites on the nanoparticles become saturated, leading to a stabilization of the adsorption process.

The findings align with previous studies, such as those conducted by Ref.^37^, which emphasize the role of nanoparticles in enhancing the adsorption kinetics of heavy metal ions. The results underscore the importance of contact duration in optimizing the removal of Pb(II) ions from solutions, providing valuable insights for future applications in environmental remediation.

The kinetics of Pb(II) ion adsorption on prepared nanoparticle samples highlight the rate of adsorption and the models used to analyze the process. The findings indicate a rapid initial adsorption phase followed by a gradual approach to equilibrium, emphasizing the role of active sites on the nanoparticles. The application of various kinetic models to better understand the adsorption dynamics. The adsorption kinetics of Pb(II) ions have been thoroughly investigated to determine the rate at which these ions are adsorbed onto the prepared nanoparticle samples. The results, illustrated in Fig. 14, demonstrate the influence of contact duration on the adsorption efficiency of Pb(II). The adsorption process can be divided into two distinct phases: an initial rapid increase in removal efficiency during the first half hour, followed by a gradual decrease as the system approaches adsorption equilibrium. This rapid adsorption is likely facilitated by the active sites present on the nanoparticles, as suggested by El-Masry et al.^38^ in their study on triphasic CoFe₂O₄/ZnFe₂O₄/CuFe₂O₄ nanocomposites for water treatment applications. These active sites enhance surface interactions with pollutants, aligning with findings from Hamdy et al.^39^ on UV photoreactor validation, were structural modifications via rare-earth doping improved adsorption efficiency.

To analyze the adsorption kinetics, three widely recognized models were employed: the pseudo-first-order model, the pseudo-second-order model, and the intra-particle diffusion model. The equations for these models are as follows:

1. Pseudo First-Order Model

14 $$\mathrm{ln}\left({\mathrm{q}}_{\mathrm{e}}-{\mathrm{q}}_{\mathrm{t}}\right)={\mathrm{lnq}}_{\mathrm{e}}-\frac{{\mathrm{k}}_{1}}{2.303}\mathrm{t}$$

2. Pseudo Second-Order Model

15 $$\frac{\mathrm{t}}{{\mathrm{q}}_{\mathrm{t}}}=\frac{1}{{\mathrm{k}}_{2}{\mathrm{q}}_{\mathrm{e}}^{2}}+\frac{\mathrm{t}}{{\mathrm{q}}_{\mathrm{e}}}$$

3. Intra-Particle Diffusion Model

16 $${\mathrm{q}}_{\mathrm{t}}={\mathrm{k}}_{3}{\mathrm{t}}^{1/2}+\mathrm{C}$$

In these equations, (k_1_), (k_2_), and (k_3_) represent the rate constants for the pseudo-first-order, pseudo-second order, and intra-particle diffusion models, respectively.

**Fig. 15:**
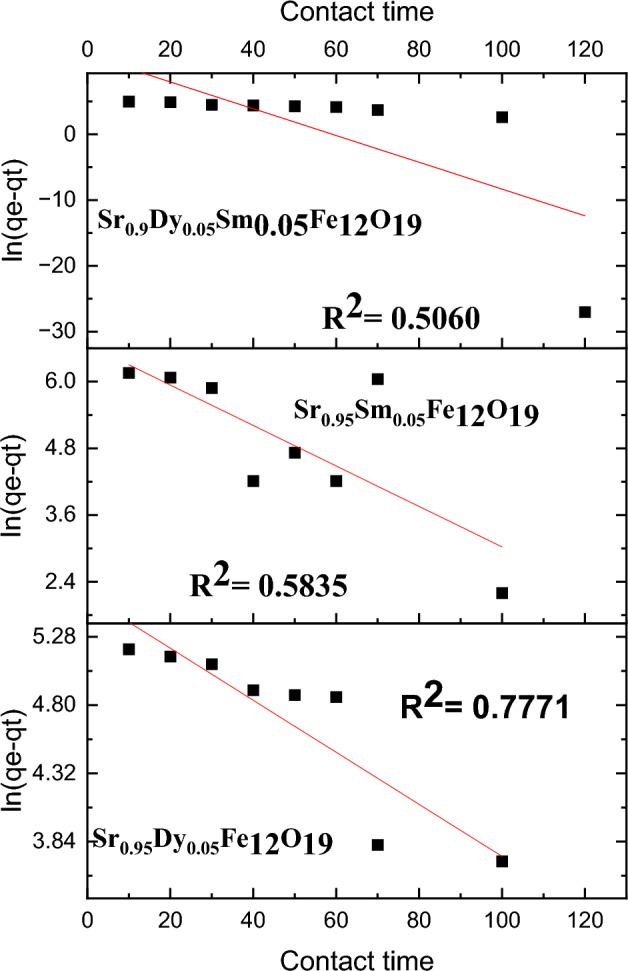
Pseudo-first-order, model for the Pb^2+^ adsorption on the surface of prepared sample.

**Fig. 16:**
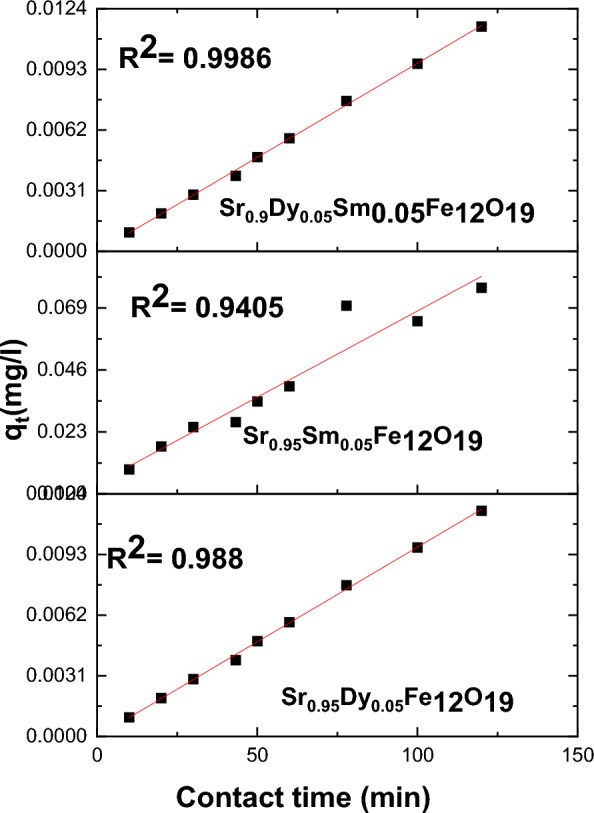
Pseudo-second-order, model for the Pb^2+^ adsorption on the surface of prepared sample.

**Fig. 17:**
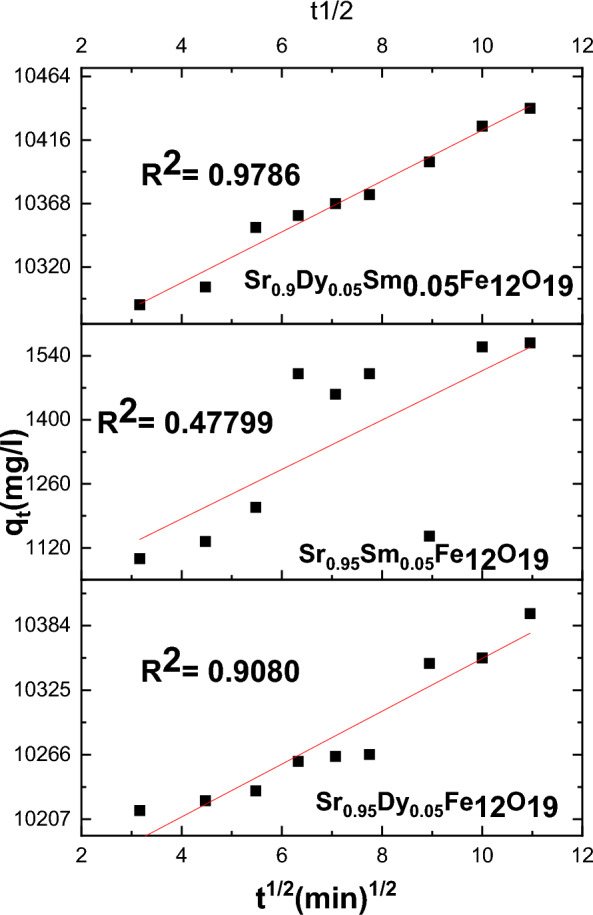
Intra particle models for the Pb^2+^ adsorption on the surface of prepared particles.

The adsorption process may represent a rate-limiting step in the chemical adsorption procedure. This is illustrated in Figs. 15, 16, and 17 where the pseudo-second-order model demonstrates a strong fit for the Pb(II) adsorption data, as indicated by the R^2^ parameters. The effective adsorption of Pb(II) can be attributed to several factors, including the surface area of the nanoparticles, the chemical nature of Pb(II), and the minimal internal diffusion resistance at the interface.

Here in, the kinetics of Pb(II) ion adsorption onto nanoparticles reveal a rapid initial adsorption phase followed by a gradual approach to equilibrium. The application of kinetic models, particularly the pseudo-second-order model, provides valuable insights into the adsorption dynamics, highlighting the significant role of nanoparticle characteristics in the adsorption process. Further studies may explore the optimization of these nanoparticles for enhanced adsorption efficiency.

#### Analysis of Pb^2+^ adsorption using Langmuir and Freundlich isotherms

**Fig. 18:**
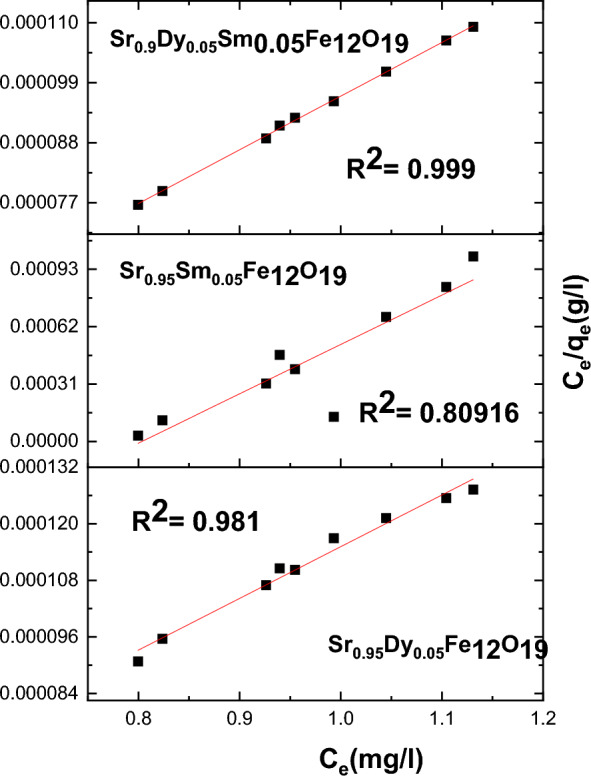
The Freundlich isotherms for the adsorption of Pb^2+^ on prepared samples.

**Fig. 19:**
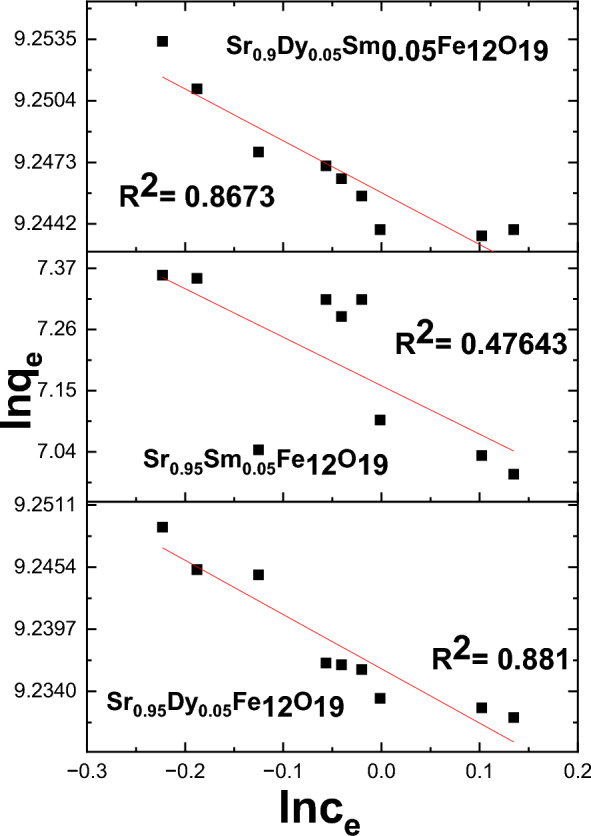
The Langmuir isotherm for the adsorption of Pb^2+^ on the prepared samples.

Figures 18 and 19 present an analysis of Pb^2+^ adsorption data utilizing the Freundlich and Langmuir isotherm models. The equations for these models are applied to evaluate the adsorption characteristics of various nanoparticles. The findings indicate that the Freundlich model provides a better fit for the experimental data, as evidenced by the R2 parameters.

The adsorption isotherms are represented by the following Eqs. (17) and (18):

Langmuir Isotherm

17 $$\frac{{\mathrm{C}}_{\mathrm{e}}}{{\mathrm{q}}_{\mathrm{e}}}=\frac{1}{{\mathrm{k}}_{\mathrm{l}}{\mathrm{q}}_{\mathrm{m}}}+\frac{{\mathrm{C}}_{\mathrm{e}}}{{\mathrm{q}}_{\mathrm{m}}}$$

Freundlich Isotherm

18 $${\mathrm{lnq}}_{\mathrm{e}}={\mathrm{lnk}}_{\mathrm{f}}+ \frac{1}{\mathrm{n}}{\mathrm{lnC}}_{\mathrm{e}}$$

According to the analysis, the Freundlich model was found to be the most effective in fitting the adsorption data, as supported by the findings of Ref.^40^.

These results indicate that multilayer adsorption occurs for the studied nanoparticles, with the Freundlich model effectively capturing the adsorption behavior of Pb^2+^ ions. The high R^2^ values suggest a strong correlation between the experimental data and the Freundlich isotherm, affirming its suitability for modeling the adsorption process in this context.

#### Reusability of hexaferrite nanoparticles in environmental decontamination

The reusability of specific hexaferrite nanoparticles in environmental decontamination. The findings indicate that these nanoparticles maintain significant effectiveness across multiple cycles of use, highlighting their potential for sustainable applications in pollution remediation.

### The ability to reuse

**Fig. 20:**
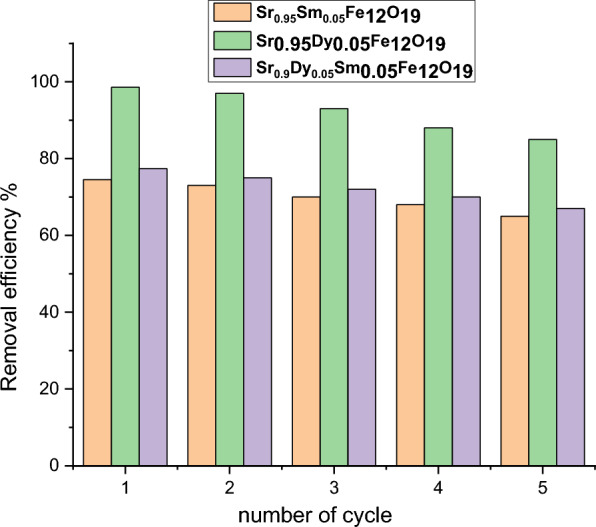
Reusability of the prepared samples for Pb (II) adsorption.

To ensure that the environmental decontamination technique is both feasible and economical, the reusability of the nanoparticles was tested over five cycles. The results of this investigation are illustrated in Fig. 20.

After four cycles, the elimination percentage of Pb (II) for each type of nanoparticle was as follows:

1. Sr_0.95_Dy_0.05_Fe_12_O_19_ - 65%,
2. Sr_0.95_Dy_0.05_Fe_12_O_19_ - 85%,
3. Sr_0.9_Dy_0.05_Sm_0.05_Fe_12_O_19_ - 67%.

These results indicate that the prepared hexaferrite samples can be effectively reused, demonstrating their viability for repeated applications in environmental decontamination processes.

#### Comparing the efficacy of prepared nanoparticles with alternative sorbents for Pb (II) ion removal

A comparative analysis of the maximum removal efficiency of Pb (II) ions using various sorbents, with a particular focus on Sr_0.95_Dy_0.05_Fe_12_O_19_ nanoparticles. The findings illustrate that the sorbents evaluated in this study demonstrate superior performance compared to previously reported materials, highlighting the significance of surface composition and physical characteristics in adsorption capacities. Comparison of Sorbents, Table 4 compiles the maximum removal efficiencies of Pb (II) ions achieved by different sorbents, including Sr_0.95_Dy_0.05_Fe_12_O_19_ nanoparticles. The data indicates that the samples prepared and evaluated in this study outperform all other sorbents referenced in the literature, including those reported in Ref.^41^. The variations in adsorption capacities among these sorbents can be attributed to differences in their surface compositions and physical characteristics. These factors play a crucial role in determining how effectively each sorbent can interact with and remove Pb (II) ions from solution.

**Table 4:** Comparing with alternative absorbents.

Adsorbent	Removal efficiency (%)
γ-Al_2_O_3_	75
Ni-doped magnetite	78
CaFe_2_O_4_	80
GdFe_0.8_Co_0.2_O_3_	40
Sr_0.95_Dy_0.05_Fe_12_O_19_	98.5

Finally, the superior performance of Sr_0.95_Sm_0.05_Fe_12_O_19_ nanoparticles in removing Pb (II) ions underscores the importance of selecting appropriate sorbent materials based on their unique properties for effective environmental remediation applications.

Table 5 provides a comparative summary of the structural, optical, magnetic, and functional properties of Sm- and/or Dy-doped SrFe₁₂O₁₉ hexaferrites synthesized in this work against recent literature. The results confirm that rare-earth doping induces porous, agglomerated morphologies, narrows the optical band gap (0.5–2.4 eV), enhances refractive index and optical conductivity, and maintains competitive magnetic performance (Mₛ: 49.3–54.1 emu/g; H_c: 3040–4899 Oe). Although reflection loss in the 1 Hz–20 MHz range is modest (− 0.15 dB), the very small skin depth at kHz–MHz frequencies suggest potential for low-frequency EMI shielding. Additionally, the materials demonstrate high Pb^2^⁺ adsorption efficiency, consistent with reports linking rare-earth doping to increased surface reactivity. Overall, the findings align well with established trends while highlighting the tunability of hexaferrite properties through strategic Sm/Dy co-doping.

**Table 5:** Comparative summary of structural, optical, magnetic, and functional properties of rare-earth-doped SrFe₁₂O₁₉ hexaferrites.

Property	This Work (Sm/Dy-doped SrFe₁₂O₁₉)	Literature Comparison	Ref
Morphology (SEM)	Agglomerated porous fragments	Similar porous, plate-like agglomerates observed in Sm-doped SrFe₁₂O₁₉ synthesized via sol–gel; porosity attributed to gas evolution during calcination	^42^
Optical Band Gap (E_g)	0.5–2.4 eV	Undoped SrFe₁₂O₁₉: ~ 1.7 eV; Sm^3^⁺ doping reduces Eg to 1.3–1.5 eV due to 4f.–3d transitions; Dy^3^⁺ induces mid-gap states, further narrowing Eg	^43^
Refractive Index (n)	Significantly increased with Sm/Dy doping	Rare-earth doping increases polarizability and electron density, raising n from ~ 2.4 (pure) to > 2.8 (doped); consistent with Lorentz-Lorenz relation	^44^
Optical Conductivity	Enhanced by Sm/Dy incorporation	Increased optical conductivity linked to f–d transitions and oxygen vacancy-mediated hopping; confirmed via UV–Vis and impedance spectroscopy	^45^
Saturation Magnetization (M_s)	49.3–54.1 emu/g	Typical Ms for Re-doped SrFe₁₂O₁₉: 45–60 emu/g; Sm^3^⁺ (non-magnetic) slightly reduces Ms; Dy^3^⁺ (high anisotropy) preserves or enhances M_s due to spin–orbit coupling	^46^
Coercivity (H_c)	3040–4899 Oe	Hc increases with RE doping due to enhanced magnetocrystalline anisotropy; Dy^3^⁺ > Sm^3^⁺ in anisotropy strength. Co-doping can reduce Hc due to lattice strain relaxation	^47^
Reflection Loss (RL)	Max RL ≈ –0.15 dB (1 Hz–20 MHz)	RL in MHz range is rarely reported; most studies focus on GHz	^48^
Skin Depth (δ)	Very small at kHz–MHz → suitable for EMI shielding	Low-frequency skin depth < 1 mm in ferrites enables thin shielding layers; Sm/Dy enhance conductivity, reducing δ further	^49^
Heavy Metal Adsorption (Pb^2^⁺)	High efficiency under optimized pH/temp	SrFe₁₂O₁₉ shows affinity for Pb^2^⁺ via surface –OH groups; RE doping increases active sites and surface area, boosting adsorption capacity (> 80 mg/g reported)	^49^

## Conclusion

The textile and dyeing industries face significant environmental challenges, particularly due to the discharge of wastewater containing heavy metals and organic pollutants. This study addresses these issues by synthesizing and characterizing multifunctional M-type strontium hexaferrites (M-SrHFs), which are engineered through auto-combustion. These materials serve dual purposes: efficient wastewater remediation and low-frequency electromagnetic attenuation behaviour.

The structural and magnetic properties of rare-earth-doped M-SrHFs—including Sr₀.₉₅Sm₀.₀₅Fe₁₂O₁₉, Sr₀.₉₅Dy₀.₀₅Fe₁₂O₁₉, and Sr₀.₉Sm₀.₀₅Dy₀.₀₅Fe₁₂O₁₉—were optimized for lead adsorption. Notable efficiency was achieved under varying conditions of pH, concentration, and temperature. Additionally, these materials exhibited tunable electromagnetic behavior. The Dy-doped variants showed superior high-frequency shielding, achieving a reflection loss of approximately -0.15 dB at 20 MHz. Co-doped samples demonstrated resonant absorption in the 3–4 MHz range, with a skin depth of about 10⁶ m at 100 mHz. The dual functionalities of these materials arise from the combined effects of dopant-induced surface reactivity, which enhances adsorption, and tailored permeability-conductivity profiles that facilitate EMI attenuation in the MHz range. Integrating nanotechnology into wastewater treatment, as demonstrated in this study, offers a sustainable solution to mitigate industrial pollution. Furthermore, the dual-purpose design of M-SrHFs addresses two critical industrial needs: reducing hazardous effluent discharge and protecting sensitive electronics from interference.

## Data Availability

The corresponding author will provide the data upon request.
